# Functional recovery time after facial fractures: characteristics and associated factors in a sample of patients from southern Brazil

**DOI:** 10.1590/0100-6991e-20202581

**Published:** 2021-01-04

**Authors:** VINÍCIUS AZEREDO MULLER, GUSTAVO KRUMMENAUER BRUKSCH, GIORDANO SANTANA SÓRIA, KAREN DA ROSA GALLAS, FLÁVIO RENATO REIS DE-MOURA, MYRIAN CAMARA BREW, CAREN SERRA BAVARESCO

**Affiliations:** 1 - Universidade Luterana do Brasil, Odontologia - Canoas - RS - Brasil; 2 - Hospital de Montenegro, Cirurgia Bucomaxilofacial - Montenegro - RS - Brasil; 3 - Grupo Hospitalar Conceição, Saúde Comunitária - Porto Alegre - RS - Brasil

**Keywords:** Epidemiology and Biostatistics, Facial Injuries, Oral and Maxillofacial Surgeons, Epidemiologia e Bioestatística, Traumatismos Faciais, Cirurgiões Bucomaxilofaciais

## Abstract

Understanding the cause, severity, and elapsed time for the restoration of the functions of maxillofacial injuries can contribute to the establishment of clinical priorities aiming at effective treatment and further prevention of facial trauma. The objective of this study was to understand the factors associated with the restoration of mastication, ocular, and nasal functions in the face of trauma victims, estimating their recovery time after surgical treatment. We analyzed 114 medical records of patients treated at the Hospital Montenegro, who attended follow-up consultations for up to 180 days. For analysis of the recovery time, we performed survival analysis, followed by COX analysis. We observed that half of the patients recovered their functions within 20 days. The average time for recovery from trauma in the zygomatic-orbital-malar-nasal complex was 11 days, and in the maxillary-mandibular complex, 21 days (HR: 1.5 [0.99 2.3], p = 0.055). Although functional reestablishment has reached high rates after the surgical approach, it is necessary to analyze the failing cases, as well as the economic impacts and the prevention strategies associated with facial trauma, to improve the service to the population.

## RESUMO

O entendimento da causa, da gravidade e do tempo decorrido para o restabelecimento das funções de lesões maxilofaciais pode contribuir para o estabelecimento de prioridades clínicas objetivando o efetivo tratamento e prevenção dos traumatismos de face. Assim, o objetivo deste estudo foi compreender quais os fatores associados ao restabelecimento das funções mastigatórias, oculares e nasais em vítimas de trauma de face, estimando o tempo para recuperação das funções, após o tratamento cirúrgico. Foram analisados 114 prontuários de pacientes atendidos no Hospital de Montenegro que compareceram às consultas de acompanhamento por até 180 dias. Para a análise do tempo para a recuperação, foi realizada a análise de sobrevida, seguida da análise de COX. Observou-se que metade dos pacientes recuperaram as funções em até 20 dias, sendo que o tempo médio para recuperação dos traumas no complexo zigomático-orbitário-malar-nasal foi de 11 dias e do complexo maxilo - mandibular de 21 dias (HR: 1,5 (0,99 - 2,3) p=0,055). Embora o restabelecimento das funções tenha atingido taxas elevadas após abordagem cirúrgicas, faz-se necessária a análise dos casos de insucessos bem como os impactos econômicos e as estratégias de prevenção associados aos traumas de face a fim de qualificar o serviço prestado à população.

## INTRODUCTION

Epidemiological studies on facial trauma are important to establish an adequate treatment approach, evaluate the ability to restore functions, and establish ways of prevention. The recovery of functions and aesthetics after the treatment of facial trauma, as well as the inconvenience of psychological wear and tear, reveal a much greater repercussion of this disease and deserves attention by health institutions^1^.

There is considerable variation in the etiology of maxillofacial trauma due to local, demographic, and social factors. It may be related to falls, sports practice, traffic accidents, violence by assaults and gunshots, and labor accidents^1^ ^,^ ^2^.

Some studies in the literature have used the concept of survival analysis to understand the impact of oral health on patients’ rehabilitation^3^ ^,^ ^4^. These have used the concept of survival analysis to understand the impact of oral health in cases of patients with head and neck cancer^3^ and the success rate of implants^4^ and dentistry^5^. Despite the large number of articles reporting the epidemiology of facial trauma, we have found no analysis of the recovery rate of these patients over time in the literature.

In this context, the objective of this study was to understand the main factors associated with the restoration of masticatory, ocular, and nasal functions in victims of facial trauma occurring in the region covered by the Oral and Maxillofacial Surgery and Traumatology Service at the Hospital Montenegro, in the period between June 2013 and August 2018, estimating the functional recovery time after surgical treatment of such traumas. 

## METHODS

In this longitudinal retrospective study, we collected data from patients sustaining trauma in the face who were treated by the Oral and Maxillofacial Surgery and Traumatology Service of the Hospital Montenegro between 2013 and 2018. The main researcher was the surgeon or first assistant during the surgical treatment. Patients underwent internal fixation of fractures with plates and 1.5 mm, 2.0 mm, and 2.4 mm screws systems, as recommended by the AO Foundation^6^, with outpatient postoperative follow-up for up to 180 days. To identify the fracture, we performed clinical examination, associated with an image exam, whether tomography or digital radiography. We excluded patients who were victims of facial trauma and did not need surgical treatment, as well as those who did not undergo postoperative follow-up.

We gathered information on the measured variables from the patients’ medical records, regarding epidemiology (age, sex), trauma (etiology, fracture region), and surgical procedure (surgical complications, type of fixation, and case monitoring). The analyzed variables associated to functional recovery were mouth opening, masticatory efficiency, and paresthesia for mandibular fractures; chewing and paresthesia for maxillary fractures; paresthesia and diplopia for orbital fractures; and anosmia and asymmetry for nose bones (NB) fractures. The time for the outcome analysis was up to 180 days, with individualized consultations according to each patient’s needs.

The analysis of the data included a census of all patients seen in the pre-determined period for data collection and who met the inclusion criteria. Based on the historical series of visits to the hospital, we expected a sample size of approximately 250 patients. We performed a descriptive analysis, calculating the frequency and the percentage for categorical variables. We applied the chi-square test for factors associated with trauma. We performed the survival analyses and the Hazard Ratio using the R software, version 3.6.0. The confidence level adopted was 95%. Thus, only analyses whose difference displayed a p value below 0.05 were deemed statistically significant. We considered all patients without functional recovery or who did not returned for evaluation as treatment failures. The present study was evaluated by the Ethics in Research Committee of the Lutheran University of Brazil and was approved under number 3,087,859. 

## RESULTS

During the period analyzed, 114 patients were seen at the Oral and Maxillofacial Surgery Service with facial fractures and met the inclusion criteria for the study (Table 1). Of these, 98 (80%) were male and 26 female (20%), with a ratio of 4:1, and a mean age of 39 years (range 7 82). Regarding the level of education, we observed that the individuals predominantly had incomplete high school or less (64%). Mandibular fractures occurred in 40 cases (35.1%), a lower rate than orbital fractures, which happened in 46 (40.4%). Trauma involving only the maxilla was limited to five cases (4.4%), fractures of nose bones (NB) represented 12 cases (10.5%), and fractures of the frontal bone, zygomatic arch, and Le Fort type I comprised three cases each (2.6%). We observed Le Fort II fractures in only two cases (1.8%) (Table 1). 

**Table 1:** Patients’ characteristics (n = 114).

Variable	Number	%
Sex		
- Female	26	20
- Male	98	80
Marital status		
- Single	41	36
- Married	60	53
- Divorced/Widowed	12	11
Types of Fracture		
- Mandibular	40	35.1
- Maxillary	5	40.4
- Orbital	46	4.4
- Nose	12	10.5
- Frontal	3	2.6
- Zygomatic	3	2.6
- Le Fort I	3	2.6
- Le Fort II	2	1.8
Etiological agent		
- Assaults	29	25.5
- Traffic Accidents (Car / Motorcycle)	28	24.5
- Falls of several types	40	35.1
- Gunshot wounds	3	2.6
- Other	14	12.3

We treated the cases according to the indication of each fracture, 57 with 1.5 mm and 38 cases with 2.0 mm internal fixation system. In addition, we used plates of the 2.4 mm system for cases of comminuted fracture of the mandible. Cases of NB fractures required contention with plaster, and in fractures to the zygomatic arches there was no need for fixation.

**Figure 1:**
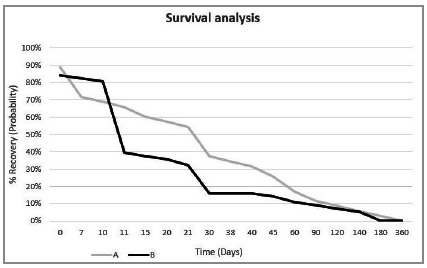
Recovery time and Confidence Interval (95%) according to facial trauma. Group A (Maxillary and Mandibular); Group B (Zygomatic, Orbital, Malar, and Nasal).

When analyzing the traumas globally, we found that half of the patients displayed functional recovery in up to 20 days. However, when stratified by fracture types, a maxillomandibular group (group A) and a zygomatic-orbital-malar nasal group (group B), the latter showed a significant reduction in recovery time (Figure 1). 

**Figure 2:**
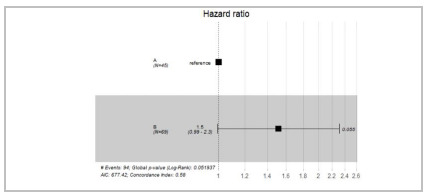
Hazard Ratio between Groups A (Maxillary and Mandibular) and B (Zygomatic, Orbital, Malar, and Nasal).

Regarding the difference between groups A and B, the Hazard Ratio in group B was higher (1.5), which pointed to a shorter recovery time, though not significant (p = 0.055) (Figure 2).

The Cox regression revealed a relationship between the time of functional recovery and the independent variables sex, age, type of fractures, and the type of etiological factor, with no identified statistical evidence (p > 0.05) (Table 2). 

**Table 2:** Relationship between functional recovery time and the independent variables. Cox Regression Analysis.

Variable	p-value
Sex	0.7976
Fracture Type	0.6847
Fracture Etiology	0.3658
Age	0.4782

To verify whether the type of fracture had a direct impact on the variables associated with functional recovery, we use the Pearson’s chi-square test to analyze the associations of mouth opening, masticatory efficiency, and paresthesia for mandibular fractures, chewing and paresthesia for maxillary fractures, paresthesia and diplopia for orbital fractures, and anosmia and asymmetry for NB fractures. We observed an association only between mandibular fracture and chewing problems (p < 0.05) (Table 3).

**Table 3:** Relationships between variables associated with functional recovery and the type of fracture.

Tested Association	p value
Mandibular Fracture × Mouth Opening	0.2329
Mandibular Fracture × Chewing	< 0.0004*
Mandibular Fracture × Paresthesia	0.6772
Maxillary Fracture × Chewing	0.0700
Maxillary Fracture × Paresthesia	0.3785
Orbit Fracture × Paresthesia	0.2655
Orbit Fracture × Diplopia	0.1645
Nasal Bone Fracture × Anosmia	0.2628
Nasal Bone Fracture × Asymmetry	0.4703

## DISCUSSION

This study is an innovation in the field of maxillofacial surgery, since there is no other found in the reviewed literature that has evaluated the recovery time of patients surgically treated after facial trauma.

Many epidemiological studies addressing facial fractures are carried out worldwide. These traumas are influenced by the socioeconomic status of the population, by educational activities, by the geographical area, and by the research period. The data we found on the patient’s characteristics are in line with the literature regarding the predominant sex and age range^1^ ^,^ ^2^ ^,^ ^7^ ^,^ ^8^.

As for the etiologic agent, recent studies associate the reduction of traumas related to automobile accidents to the mandatory use of seat belts and the fact that airbags have become a mandatory factory item in all cars, currently overcome by trauma associated with interpersonal violence^8^. Regarding motorcycle and bicycle accidents, the use of open helmets or helmets without a chinstrap increases the likelihood of facial trauma resulting from inadequate protection^9^. We should note that falls from standing height are normally associated with advanced age, and campaigns preventing falls in the elderly are necessary^6^ ^,^ ^7^.

 In the present study, the classification of fractures and types of complications are in agreement with the data described in the literature, reporting that in Le Fort fractures there is split between the pterygoid plates and the posterior maxilla, and can be classified into three types, which are associated with various combinations of fractures and clinical complications. In addition, the presence of a naso-orbito-ethmoid lesion depends on the extent of the lesion to the fixation of the medial cantal tendon, with possible complications such as rupture of the nasofrontal duct, while fractures of the zygomatic-maxillary complex can trigger an increase in orbital volume and enophthalmos. In orbital fractures, injury to the lower rectus muscles can lead to diplopia or damage to the eye globe or infraorbital nerve. Fractures of the frontal sinus that extend through the posterior sinus wall can create communication with the anterior cranial fossa, resulting in leakage of cerebrospinal fluid and intracranial bleeding^10^.

 In this context, in the present study only the act of chewing displayed functional alterations, with difficulty in opening the mouth, painful symptoms, abnormal mandibular movements, malocclusion, and edema^11^. We should note that due to the small sample size, some associations could not be perceived; hence, the need of larger samples studies to confirm these results. In addition, the region of the country and the hospital’s characteristics may have influenced results, requiring multicentric analyzes to confirm the presented data. We treated the patients included in this study with internal fixation associated with open fracture reduction, applying plates and screws, as recommended by the AO Foundation for each type of face fracture. This is the more acceptable method, providing the best stability results and greater accuracy due to the reduction of the fragments^9^ ^,^ ^12^. In this context, Lee (2009)^13^ demonstrated that of the patients treated for facial trauma, 59% were hospitalized and 56% required surgery, with 41% internal fixation.

When analyzing the functional recovery of patients after surgical treatment, we observed that half of the patients recovered from the sequelae of facial trauma within 20 days after surgical treatment. Of the entire sample, nine patients did not completely recover within 180 days and were classified as treatment failure. These patients presented paresthesia as the treatment failure.

In the Cox regression, the results indicated no influence of the variables sex, etiological factor, age, and fracture site on the recovery time. Although there was no statistical significance for the etiological factors evaluated, in the literature there is a higher incidence of systemic complications during treatments of higher-energy traumas, such as traffic accidents and gunshot wounds^14^ ^,^ ^15^, which could suggest a longer recovery time or permanent sequelae. In addition, as for the two most predominant etiologic factors in the present study, assaults and traffic accidents, the literature indicates that men are more affected than women^16^ ^,^ ^17^. However, the lack of association in our series may be related to the sample size.

Despite not measured in this study, the economic impact of trauma to health services is quite significant. Thus, as perspectives, it is necessary to assess the impact of facial trauma regarding costs with medications and in reducing individual gain due to lost working days, relating them to the types of trauma and the associated etiological factors. As it is a pioneering analysis, further studies on the rate of functional recovery are needed.

## CONCLUSIONS

This study is an innovation in the field of Oral and Maxillofacial surgery, since no other has been found in the literature reviewed to date that evaluated the recovery time of patients surgically treated after facial trauma. We could observe that mandibular injuries are associated with a recovery time almost twice as long as the one of maxillary lesions.
